# Adolescents’ experiences of a whole-school preventive intervention addressing mental health and nonsuicidal self-injury: a qualitative study

**DOI:** 10.1186/s12889-024-20832-y

**Published:** 2024-12-02

**Authors:** Erik Aspeqvist, Ann-Charlotte Münger, Hedvig Andersson, Laura Korhonen, Imke Baetens, Örjan Dahlström, Maria Zetterqvist

**Affiliations:** 1 Center for Social and Affective Neuroscience, Department of Biomedical and Clinical Sciences, Linköping University, Linköping, Sweden; 2 Barnafrid, Swedish National Center on Violence Against Children, Department of Biomedical and Clinical Sciences, Linköping University, Linköping, Sweden; 3 Department of Child and Adolescent Psychiatry, Region Östergötland, Linköping, Sweden; 4 Brussels University Consultation Center (BRUCC), Department of Clinical Psychology, Vrije Universiteit Brussel, Brussels, Belgium; 5 Department of Behavioural Sciences and Learning, Linköping University, Linköping, Sweden; 6 Athletics Research Center, Linköping University, Linköping, Sweden

**Keywords:** Adolescents, Mental health, Intervention, Nonsuicidal self-injury, Self-harm, School, Community sample, Thematic analysis

## Abstract

**Background:**

Programs for mental health promotion and prevention of nonsuicidal self-injury (NSSI) in schools have gained increased focus during the last decades, but less is known about adolescents’ experiences of such interventions.

**Methods:**

A whole-school preventive intervention targeting mental health and NSSI was delivered to six secondary schools. Adolescents participated in the Youth Aware of Mental Health program combined with an NSSI-focused psychoeducation module. Caregivers and teachers were given online psychoeducation on NSSI, and school health care staff were given a workshop on self-injury. Eleven group interviews (*n* = 65 participants) were conducted with adolescents (ages 13–15 years, 65% females) exploring participant experiences. Interviews were analyzed using thematic analysis and interpreted in light of a biopsychosocial understanding of adolescence.

**Results:**

The analysis generated two main themes. The first theme, *Mental health in the context of adolescence*, centered around adolescents’ conception of mental health, after having taken part in the intervention, framed in a context of coping with external stressors. The dilemma of autonomy versus help-seeking was also identified as part of the first main theme. The second theme, *The who*,* when*,* what*,* and how of the intervention*, described adolescents’ experiences of the intervention. This theme included increased awareness and knowledge of mental ill-health. The participants generally agreed that the topics included are important to adolescents and emphasized that the content needs to be relatable. Several factors that influence how a school-based program is received by adolescents were identified, such as who should be targeted and when. Adolescents also identified challenges and gave recommendations for future similar projects.

**Conclusions:**

Adolescents generally perceived addressing mental health and NSSI in schools as important. Help-seeking initiatives need to be balanced against adolescents’ need for autonomy when planning mental health prevention and intervention.

**Supplementary Information:**

The online version contains supplementary material available at 10.1186/s12889-024-20832-y.

## Background

Raising concern, an official Swedish public health report from 2023 identified increased rates of various mental health-related symptoms among adolescents [1]. The data is well in line with the international literature [2] and knowledge that half of lifetime mental disorders have their onset before the age of 20 [3]. An early intervention paradigm for youth mental health promotion has been proposed as a way of tackling the increased mental ill-health [4]. In the 2020 publication *Guidelines on mental health promotive and preventive interventions for adolescents* [5], for example, the World Health Organization (WHO) panel of experts recommends that psychosocial interventions should be made available to all adolescents. In Sweden, the proposed national strategy for mental health by the Public Health Agency [6] includes “increased focus on health promotion and preventive work in school” (p. 9).

One prevention program listed in the WHO guidelines is Youth Aware of Mental Health (YAM) [7]. YAM was developed with general mental health and suicide prevention in mind and is delivered to adolescents in schools. In a large multi-center study, YAM reduced suicide attempts and severe suicide ideation in school-aged adolescents [8].

Nonsuicidal self-injury (NSSI) is often mentioned in connection with adolescent mental health. NSSI is defined as the intentional destruction of one’s bodily tissue without suicidal intent and for reasons not culturally sanctioned [9]. It typically starts in early adolescence and is conceptualized as a coping strategy [10, 11], most commonly engaged in to regulate distressing emotions [12]. It is therefore differentiated from self-injurious behaviors performed with suicidal intent. Difficulties with regulating emotions have been associated with higher risk for NSSI [13] and found to mediate more distal causes of NSSI [14].

Meta-analyses have estimated the prevalence rate in adolescents to be 17–18% [15, 16]. However, recent research suggests that NSSI might be even more common, as a general survey question, which is often used in prevalence studies, might systematically fail to identify all groups of people who engage in self-injury [17]. Given the high frequencies, today´s adolescents are very likely to come across NSSI directly or indirectly.

There has been an increased interest in prevention and addressing NSSI in schools during the last decades [18, 19]. One example of a prevention program is the Signs of Self-injury [20], which has been successfully piloted and found feasible without iatrogenic effects. In a 2020 study, Baetens and colleagues piloted another prevention program with an included module on NSSI, called KRAS, with a sample of 651 secondary school students (age 11–15 years, *M* = 12.85) [21]. The program was found to be feasible in a school setting, and without iatrogenic effects. Besides investigating outcomes quantitatively, the study included a qualitative evaluation and responses were divided into “cognitive” and “emotional” categories in the analysis. The authors concluded that the addition of an NSSI-specific module may potentially have positive effects specifically for those with an NSSI history, with positive influences on help-seeking behavior.

In a more recent paper, Baetens and colleagues [22] also found positive results for a preventive program delivered in schools that targeted psychological complaints and NSSI in 11–14-year-olds. Preventive programs focusing on NSSI have also been evaluated in Italy [23] and Iran [24] with promising results. However, aside from the 2020 study by Baetens and colleagues [21], none of the interventions with NSSI-specific content have been studied using qualitative research methods to explore participant experiences.

The only qualitative study focusing on YAM participants was carried out by Wasserman and colleagues [25]. The authors identified five typical engagement patterns concerning YAM expressed by their interviewees: Engaged, Initially hesitant, Eager to please, Cautious and Disengaged, and concluded that it is important to acknowledge the existence of such heterogeneity among the participants when working with mental health promotion.

As mentioned above, adolescence has been identified as a key period for mental health promotive and preventive interventions. Thus, the transformational developmental period of adolescence needs to be considered as it is the context in which such interventions are deployed. Adolescence is a transitional period between childhood and adulthood that begins with the onset of puberty and is generally considered to end at or before 20 years of age. It is characterized by biological, psychological and social advancement, with rapid development of the body and the brain specifically [26]. As social roles are in flux, adolescence is an important developmental phase for processes related to identity and autonomy [27]. Researchers have been trying to articulate what defines adolescent autonomy, and today it is generally considered a multi-faceted phenomenon [28]. Further, maladaptive identity functioning in adolescents has been associated with general psychopathology [29], and NSSI specifically [30, 31].

In adolescence, some individuals also face increasing challenges with regulating emotions [32]. Difficulties with emotion regulation have gained attention as a potential underlying, transdiagnostic factor explaining a range of mental health problems [33, 34]. In light of this, it has been suggested that interventions focusing on emotion regulation could be effective for different forms of psychopathology [35], including NSSI specifically [36].

In health promotion, encouragement of help-seeking is a common component, not least when it comes to mental health. Research suggests that a large share of the population who could potentially benefit from mental health services do not seek help even when such services are available and free of charge [37]. Lack of disclosure and help-seeking also apply to NSSI specifically, where one-third to half of adolescents with NSSI do not seek help and clinical services are rarely the preferred support system [38]. This has contributed to an increased interest in studies on help-seeking as a construct of its own, as well as factors that exert influence on help-seeking [39–43].

Although there has been rising research interest in evaluating universal mental health interventions for adolescents in school settings, few studies have given voice to the participating adolescents. In particular, there is a need for more qualitative studies on participant experiences related to NSSI prevention programs. This knowledge gap must be addressed to foster further development of mental health initiatives directed at adolescents and ways to deliver them.

### Aim and research questions

The overarching aim of the current study was to explore adolescents’ experience of a whole-school preventive intervention addressing mental health and NSSI. Specifically, the study focused on the following research questions:

- How do adolescents perceive mental health and nonsuicidal self-injury?
- How do adolescents experience a whole-school preventive intervention that addresses mental health and nonsuicidal self-injury?
- What kind of support do adolescents perceive is needed for mental health and self-injury in the educational setting?

## Method

The current qualitative study is part of a larger project where the overarching aim was to investigate the effects of a universal mental health preventive intervention that also targeted NSSI [44]. This qualitative study followed the Standards for Reporting Qualitative Research (SRQR) [45] and was carried out by a research group with experience, including but not limited to the fields of child and adolescent mental health and NSSI.

Five of the authors (EA, HA, IB, LK, MZ) have a background in clinical work with children and adolescents’ mental health and academia. EA, HA and MZ as clinical psychologists, IB as a family therapist, and LK as a child and adolescent psychiatrist. OD holds a PhD in disability research and is a senior associate professor in psychology with focus on health and sports, and AM is a qualitative researcher with a research background in mental health and school-related topics. All authors have previous experience in qualitative methodology. The research group consists of two males and five females.

### Participants and procedure

**Fig. 1:**
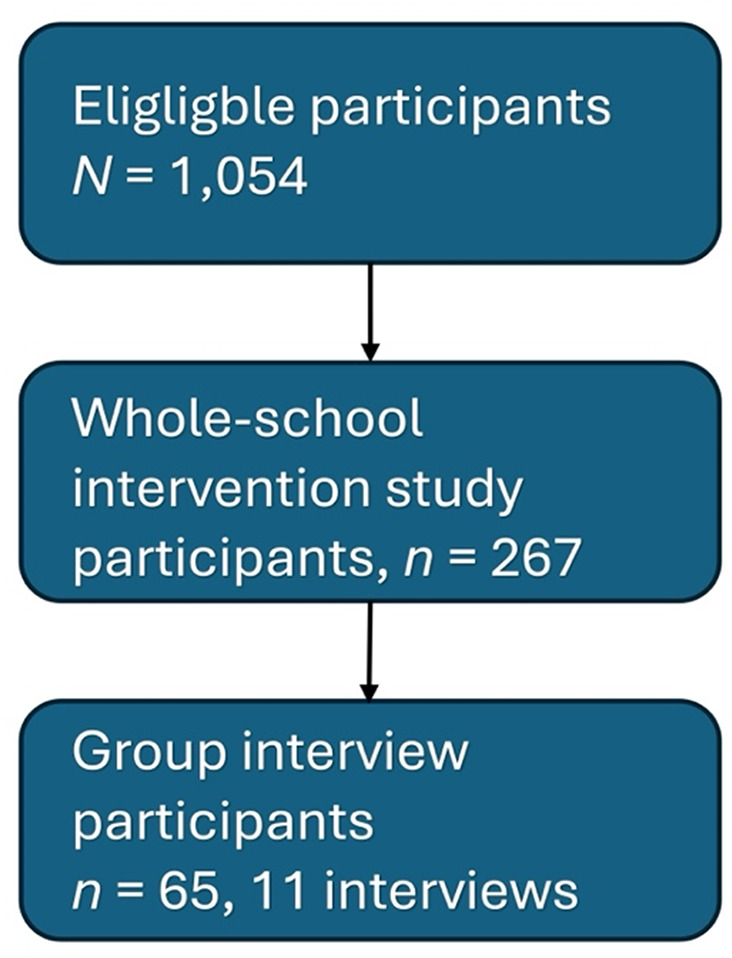
Participants’ flowchart

In the first recruitment stage of the overall school intervention study, schools were invited to participate by contacting principals of schools around Östergötland county, Sweden. Headmasters from seven schools responded and proceeded to the second stage where students and their caregivers received written information about the project and were invited to participate. It was decided not to include participants at one of the participating schools as it did not reach a participation rate above 5%. Written informed consent was required from both the students themselves and their caregivers. This resulted in 267 out of 1,054 eligible students participating in the overall study (25.3% participation rate; see Fig. 1). Eligible students were those in a standard (i.e., not a special education class for students with intellectual disability or a class for newly arrived refugees with limited knowledge of the Swedish language) grade seven or eight school class, following the standard Swedish curriculum. Participating students in the overall study were between 13 and 15 years old (*M* = 14.21, *SD* = 0.56), and 58.3% were female.

The preventive intervention delivered to the participating schools was carried out in 2022. Schools were randomized to intervention or control condition, and those in the control condition received the same interventions at a later stage. The preventive intervention included components directed at students, parents and school staff. In class, students participated in the Youth Aware of Mental Health (YAM) [8] prevention program. YAM combines psychoeducation with experiential learning through role-play during five hours of classroom-based sessions over three weeks. Students also participated in the KRAS [21] single-session workshop on NSSI, which was translated into Swedish. Additionally, school psychologists, nurses and counselors, were offered a two-day on-site interactive workshop on NSSI and suicidality based on translation and adaptation of the 4S (Strong Schools Against Suicidality and Self-Injury) [46, 47] program developed in Ulm, Germany. Parents and teachers were offered a web-based psychoeducation workshop with specific information about NSSI developed by the research group.

After the completion of the overall preventive intervention study (*n* = 267 for all the schools), participating students were invited to voluntarily participate in group interviews about their experiences of the intervention. Sixty-five students who had provided informed written consent with consent from the caregivers were included. Two interviews, one per grade (7th and 8th), were conducted at all schools, except for one school where only one was done, resulting in 11 interviews. The groups consisted of 3–7 adolescents (65 total participants; in eight of the interviews, six adolescents participated). A balance between girls and boys was aimed for; the proportion of females in all the interviewees was 65%. Besides gender, no other data was collected in connection with the interviews.

The study was approved by the Swedish Ethical Review Authority (2021 − 01699, 2021–05049).

### Data collection / interviews

EA and HA performed the group interviews, in some cases with help from two research assistants. The lead interviewer (either EA or HA) was accompanied by a supporting interviewer in an observational role. Interviews were carried out at schools and were audio-recorded. Interviews were semi-structured and followed an interview guide developed specifically for the present study (see Supplement 1). The main topics covered in the interview guide were participants’ experiences of the universal whole-school preventive intervention, what they had learned and perceived as the take-home messages, and their recommendations for targeting mental health and NSSI in schools. The interviews lasted between 23 and 57 min (*M* = 38 min, *SD* = 10 min).

### Analysis

Thematic analysis (TA) was employed with the methodological framework developed by Braun and Clarke [48, 49] in mind. The nature of the research questions and the placement and purpose of the study within the overall project led the authors to a position in the middle of the descriptive-interpretative continuum. The interview guide was put together to elicit both descriptive-level and broader answers. Some flexibility was thus allowed in the analysis, sometimes leaning toward the summarized topic descriptive while also opening up for interpretation beyond the semantic level.

Before analysis, the interview audio recordings were transcribed verbatim. Broadly following the analysis steps proposed by Braun and Clarke [49], EA, AM and MZ read through the transcripts to familiarize themselves with the data, and met several times to compare codes and generate overall themes. Overarching themes and names of themes were discussed with and approved by all authors. Quotations used in the present manuscript to highlight study findings were drawn from all interviews and translated into English.

## Results

**Table 1:** Themes

Overarching themes	Themes
Mental health in the context of adolescence	Coping in a stressful context
	Autonomy vs. help-seeking dilemma
The who, when, what, and how of the intervention	For all or for a few
	Window of opportunity
	Lessons learned
	Challenges targeting mental health in the educational setting

Two overarching themes were generated in the analysis: *Mental health in the context of adolescence*, and *The who*,* when*,* what*,* and how of the intervention*. Themes and subthemes are presented in Table 1.

### Mental Health in the Context of Adolescence

This theme describes adolescents’ perceptions of mental health in the context of adolescence after having taken part in the intervention. It contains two subthemes: *Coping in a stressful context* and *Autonomy vs. help-seeking dilemma*.

#### Coping in a Stressful Context

When adolescents talked about *mental ill-health*^1^, they returned to their perceptions that two main factors contributed to it, social media and school-related stress. This means that mental ill-health was explained in terms of a stressful context affecting everyone, and not in terms of individual experiences. One participant said that “you get like stressed, really stressed sometimes because it is a lot in school […] and then you can get a little, like, stressed and feel bad because of that”. A busy schedule with many exams was brought up several times. Concerning the influence of social media, both the pressure of living up to skewed ideals and the time consumption were also brought up: “you put a lot of time into it […] when you get stressed it is easy to, get upset […] and then you look at your phone for, like, comfort”.

Mental ill-health was characterized by the adolescents as a consequence of not being able to cope effectively enough with pressure and stress. Consequently, they reported that interventions that target mental health need to include strategies for dealing with stress related to school and social media. This was interpreted as an awareness of available strategies as well as a sense of agency and feeling responsible for your situation.

The participants generally perceived mental ill-health as being common in adolescents. Their discussion also pointed to an awareness of the fact that mental ill-health can be concealed to others, and related to stigma: “This I’ve seen a lot on [social media], where a person maybe starts talking about [nonsuicidal] self-injury […] but then you see comments where […] ‘you haven’t got any problems’, like hate-comments”.

#### Autonomy vs. Help-Seeking Dilemma

The adolescents gave voice to a dilemma, which was expressed as, on the one hand, recognizing the importance of seeking help and talking to adults about important matters. “Yes, how important it is to get help, like, and also that it is important to tell an adult if you notice something about someone else, like, a friend. You must tell someone”. This was contrasted, on the other hand, by adolescents’ perceptions that adults tend to exaggerate and easily become over-involved, which is why talking to friends sometimes was the preferred choice. One said: “I think it will be taken further and turn into […] a much bigger thing if you take it up with an adult”.

In some cases, participants also described a lack of confidence or trust as a barrier to help-seeking: “I think that counselor don’t get what you mean because they don’t know you”. Knowing where to turn for help, the availability of such help and for that person or organization to be sensitive and responsive were expressed by several as important:

I just think that it is important to people to get to know they can turn to places. I think that is very important, not that they have to but that it is important that they know they are there.

Within this dilemma subtheme, a recurrent topic expressed by adolescents concerning seeking help from adults was a wish to try to solve problems by oneself first and the importance of employing coping skills, hinting that this is an individual responsibility. ”I think you have to find it out yourself […] give it time and try to work on it yourself […] try different things and choose what feels best for yourself”.

At the same time, adolescents wanted adults, including school staff, to be available and attentive to mental health issues in adolescents: “They have to notice when the alarm bells sound”. The tension became salient, between on the one hand valuing autonomy, independence and taking care of problems yourself, and on the other hand also realizing the need for a safety net consisting of adults. Taken together this was interpreted as an Autonomy vs. help-seeking dilemma.

### The Who, When, What and How of the Intervention

The adolescents generally declared that it is important to address mental health problems in the educational setting. Overall, they were positive about receiving information, knowledge, and tips. There were, however, different perceptions of the intervention among the adolescents. Some were very appreciative and found it meaningful, while others found the intervention uninteresting and even boring or had a hard time remembering and needed several probes to recall the content. The adolescents emphasized that the intervention needs to be relatable and delivered in a way that helps to sustain attention. Further, they reflected on the pros and cons of a universal vs. targeted approach and discussed different challenges related to targeting mental health in an educational setting. Taken together this was conceptualized as *The who*,* when*,* what and how of the intervention* and is elaborated on below in the four subthemes: *For all or for a few*,* Window of opportunity*,* Lessons learned and Challenges targeting mental health in the educational setting.*

#### For All or For a Few

This subtheme contains adolescents’ experiences of for whom the intervention is most suitable, and more specifically whether an intervention addressing mental health and NSSI in an educational setting should be delivered to everyone or to those experiencing problems. On the one hand, several adolescents expressed that everybody needs information about mental health: “Everyone. Everyone needs this information.”

On the other hand, if the content was not experienced as relatable, adolescents reported that they lost interest “You would have listened a little bit more if it was true for you, now that it isn’t, I let it go”, and some thus argued for the advantages of focusing on those who need it: ”Maybe if you feel bad then it might be helpful to participate in this course, […], but I don’t think that for a normal teenager, it will make a difference.”

Further, the adolescents described that school staff and parents need information and education on mental health in adolescents. The general message was that they were positive to the inclusion of parents and school staff and the whole-school approach. ”Great that all [parents, teachers] can learn. Maybe it can help, they know how they can help […] a teacher might recognize […] if they note that a student is not feeling well.” This was especially related to the topic of social media, where the participants expressed that adults today do not fully understand the situation for adolescents in the 2020s, with the challenges and benefits of social media, such as TikTok and Snapchat: ”Yes, they have also been teenagers, but maybe it has been like more special for us in a way with, like, the internet and online hate and such things, that they maybe haven’t gone through a lot.” This also included the topic of NSSI: ”In their time there wasn’t as much about self-injury, like, it wasn’t as common.”

#### Window of Opportunity

Adolescents reflected on when preventive interventions for mental health and NSSI should preferably be delivered, and whether there is an optimal time during adolescent development to receive this information. They reported that NSSI and mental health problems usually start earlier than in seventh or eighth grade (13–14 years of age in Sweden): “It was, like, in sixth grade when, when I started hearing and seeing, on social media for example, about [nonsuicidal] self-injury and depression and things like that”, and therefore argued that an intervention needed to be delivered before junior high, typically at age 11–12: ”Because some maybe already have gone through it and then they won’t know until after.”

On the other hand, some concerns were raised that if delivered at too young an age, the content would not be relatable and too difficult to comprehend. Life experience and cognitive and emotional maturity were mentioned as important components to consider. The adolescents were concerned that if mental health problems were addressed at too young an age, there would be a potential risk that the subject would not be approached respectfully. Some suggested starting early on and building on as adolescents grew older: “You can have it a little now and then, […] taking one step at a time.”

#### Lessons Learned

When discussing the intervention, the participants reflected on what they had learned. The importance of seeking help and communicating with others about mental health problems was perceived as a take-home message. Adolescents also reported increased knowledge and understanding of how to identify potential mental health problems, both in themselves and in peers. This was analyzed as Lessons learned.

I can say that the course actually helped me to find out how I feel, I have felt mentally bad and I still do, but it really helped me to understand that I maybe need help from someone, and that I maybe need to open up for example to my parents, because I have never done that and really have a hard time opening up to people.

The participants also appreciated tips on how to help peers:

Our friend, this with [nonsuicidal] self-injury, we’ve had a friend who has […] but we have learned a little how to, take care of that. […] Sometimes she comes and, ‘I like did it again’ […] We have learned that you shouldn’t be too much in her face but that you still should try to help her in some way. […] I think some have learned a lot more about such things, or at least I have.

Some adolescents also expressed that they had become more aware that others may be feeling something other than what they display and that this new perspective in some cases had led to increased compassion and tolerance for others.

I think, courses like this, I think people get more tolerant, like, if they think before that people with mental ill-health are just whining and bla bla, but then if you take part in a course and actually learn about what it is and learn that these persons have a really hard time […] then I think you treat them in a better way.

They also discussed that adolescents could have more hope that others would understand their situation with new knowledge and that adolescents did not have to think that they were all alone if they experienced mental health problems. ”People who have a hard time maybe understand that there are people who care about you, yes, and like, that people listen. And that they’re not alone in it.” This was also expressed specifically concerning NSSI. “Then maybe persons with [nonsuicidal] self-injury can open up more and feel I need help, can someone help me, like, without needing to get yelled at.”

It is worth mentioning that a few adolescents had also perceived a more normative take-home message, that one should not engage in self-injury and to be happy instead of sad.

#### Challenges Targeting Mental Health in the Educational Setting

In addition to the lessons learned reported above, most adolescents also described challenges related to delivering mental health interventions in the classroom. The most common obstacle reported was that the subject was not always discussed respectfully by some adolescents who did not take the matter seriously. “There are people who aren’t that mature, who think that everything can be joked about.” The adolescents, however, also discussed that even those who, at first appearances, did not seem to take it seriously, probably did: “[…] there are some who, like, don’t take anything seriously. […] but I think, on the inside, they take it in.”

Thus, the climate in the classroom was a central aspect of how the intervention landed. In this context, concern was voiced that negative comments from peers could increase stigma for those who struggle with mental health problems. Some therefore argued for the advantage of targeting mental health and NSSI in smaller groups, but there were also advantages of the whole class approach. “It would probably have been easier in small groups, but it feels like the class was more involved as a whole.” The adolescents also mentioned pedagogical challenges related to sustained focus and attention. Taken together this was interpreted as Challenges targeting mental health in the educational setting.

## Discussion

In this study, we have analyzed the participant experiences from a whole-school mental health and NSSI intervention provided for adolescents. The analysis generated two main themes, one centered around adolescents’ perception of mental health and one around the intervention. Participating adolescents framed mental ill-health in a context where individual coping with external stressors was the central dynamic, focusing especially on social media and school stress. They also emphasized autonomy at the same time as acknowledging the importance of help-seeking, which was interpreted as a dilemma between the two. The participants described an increased awareness of how widespread mental health problems are and of the struggle that affected adolescents are faced with. They pointed out that they thought the topics included were important, and discussed factors influencing how a prevention program is received, such as the timing and setting. Also, participants said they understood the importance of seeking help and of offering support to peers as central messages.

Participants described that the main reason behind mental ill-health is a context where a lot of pressure is put on a person. The pressure was described to be generated, for example, by schools with constant high demands regarding performance and results, and by social media. This is consistent with results reported by Spencer and colleagues [50], who concluded that academic pressure was an important factor behind mental ill-health. The use of smartphones and social media has increased dramatically among adolescents in recent years and is associated with worse mental health [51, 52]. Recently, this has gained increased focus, internationally through the publication of Jonathan Haidt’s *The Anxious Generation* [53] and locally in Sweden through a 2024 report by the Public Health Agency pointing to risks associated with excessive use of smartphones and social media [54].

The role of social media in relation to NSSI has also gained increasing attention during the last decade, and findings support an association between problematic social media usage and self-injury (irrespective of intent) [55]. Further, positive (gaining support) and negative (being harassed) effects of social media on NSSI have also been documented [56, 57]. Another recent study, including 680,000 adolescents between 2002 and 2018, also found the increases in academic pressure and social media use to be significantly associated with increased psychological complaints [58].

Previous research related to external stressors aligns well with what was expressed by participants in the present study. In general, the adolescents did not question these conditions but described individual coping skills as key to staying afloat. They also wanted strategies for school stress and social media to be included in interventions that target mental health and NSSI. Recent intervention studies have in fact included such components [21]. From a critical point of view, the primacy of individual conceptualization of and solutions to mental health issues have been suggested to be signs of an ongoing medicalization process [59] leading to a “responsibilisation” of the individual [60].

The participants expressed several considerations regarding autonomy, support and help-seeking. We interpreted this as a strong desire to stay in control and decide for themselves when and how they would seek help. At the same time, participants expressed a wish for available, responsive, and knowledgeable adults. This was interpreted as an autonomy vs. help-seeking dilemma, which can be related to the pivotal process of separation-individuation in adolescence [61]. In the conception of adolescent autonomy, proposed by Beyers and colleagues [28], the four separate constituents are Connectedness, Separation, Detachment and Agency. The authors suggest that the most favorable situation would be a balance between Connectedness and Separation, but with a lower degree of Detachment and a higher degree of Agency. A central point to this model is that Separation, characterized by interpersonal independence, is not identical to Detachment, which is more associated with mistrust. Connectedness, on the other hand, is described as having to do with trust and the availability of the parent. In the present study, what the participants had to say regarding these matters fitted quite closely with the model by Beyers et al. [28]. Similarly, in the meta-synthesis by Roberts and colleagues [43], the need for autonomy was found to be the central theme drawn from all the included studies. The authors pointed out that regarding therapy, “[a]dolescents need to use their autonomy to choose when they engage, who they engage with, what is done in therapy and what type of therapy they receive” (p. 123) and that systemic barriers as well as mental health literacy can act as barriers to adolescents’ utilization of mental health services. Previous findings that stigma and trust issues as well as lack of knowledge and practical matters, such as availability and locations [41, 42] were also evident in the interviews in the present study.

Concerning participants’ experiences of the whole-school preventive intervention, the overall message was that they considered it to be important. Several stated that interventions like these should be made available to all adolescents, and the adolescents discussed pros and cons regarding the right time for delivering them. The fact that the whole-school approach meant that school staff and parents were also included was especially appreciated by the adolescents. However, some participants explained that they needed the content to feel relevant to find it meaningful. This was presented as a challenge that could partly explain the fact that the intervention, in their opinion, was not taken seriously by everyone. This, in turn, was a factor that influenced the experience of the whole class. Another potential challenge when delivering mental health interventions is that adolescents might perceive a normative message different from the intended one [62], which was mentioned by a few of the study participants.

The fact that adolescents reported that their peers engaged with the material to a varying degree echoes what was found by Wasserman and colleagues [25] in the only previous qualitative study on YAM participants. Thus, we agree in concluding that it is important to acknowledge that adolescents will engage with mental health promotion programs to a varying degree. Likewise, Baetens and colleagues [21] sorted adolescents’ experiences into a tree-structure, underlining the point that participants at the individual level respond to an in-class prevention program in a wide range of ways.

When discussing their experiences, adolescents also reflected on who should be targeted. Interestingly, tapping into the pros and cons of universal vs. targeted preventive approaches, adolescents identified the importance of reaching out to everyone and simultaneously pointed to the challenges when the material is not perceived as relatable for everyone. In fact, the issue of who interventions are best delivered to is a central topic in the research field of prevention [63–65]. Some adolescents who reported that they felt that the content was not for them, unknowingly expressed what has been referred to as the Prevention paradox – the fact that for universal prevention to reach as many as possible of those who can benefit from it, many who will be less likely to benefit will have to participate as well [62, 66]. This potentially also applies to participants who initially reported not remembering much of the interventions and who needed further probes in the interview. This could be a sign that they had not actively engaged with the material. Further, it has been suggested earlier that the use of negative content and psychiatric terminology might induce a feeling of distance from the target audience in some participants [62, 67].

The adolescents in the current study reflected on the best time to deploy a mental health preventive intervention. There was agreement that the content they had received was important to adolescents. The adolescents argued that it is important not to be too late if the goal is prevention, but on the other hand, at an earlier age fewer students might be receptive to the material. The age of onset for NSSI has shown to be around 11–13 years in recent studies of Scandinavian populations [17, 68] and the onset of mental health problems usually coincides with the onset of puberty [69]. Heath et al. [70], for example, emphasize the importance of conducting the intervention right before the development of the behavior that one wants to target. There are programs delivered in middle/secondary school targeting 11–14-year-olds with promising effects, reducing NSSI and psychological distress, indicating that it is feasible and acceptable to target al.so younger age groups [22].

Several participants mentioned increased insight and awareness of the fact that you cannot necessarily sense how another person feels and that many might struggle with things they keep on the inside. This aligns with findings by Baetens et al. [21], who reported that some participants expressed an increased sense of empathy towards those who engage in NSSI. Increased knowledge and awareness through education has been shown to improve attitudes and reduce stigma concerning mental health in general [71] and NSSI specifically [72]. Participants also talked about how they had perceived a strong message about the importance of seeking professional help if the situation was more serious.

Like with other mental health-related topics discussed, adolescents described an increased awareness of the fact that NSSI is common, yet might often be concealed from peers, as well as family and other adults. This can be interpreted as a better understanding of the stigmatization and struggle that young people engaging in NSSI are faced with [73]. Some participants also expressed being more comfortable with how to treat others if they disclosed personal NSSI experience as well as some hopeful thoughts that this might lead to an increased openness about it. This is important, seeing that adolescents and young adults tend to disclose NSSI primarily to peers [74]. However, some participants explained that they felt NSSI, and related topics were something they had a hard time relating to and therefore did not engage with the material.

In general, the participants were not initially very expressive when it came to describing their experience and what they had learned from the YAM and KRAS sessions, and some of them said they hardly remembered participating at all. Notwithstanding, looking at the whole material and the themes that were the results of the analysis, the prevention program might have influenced adolescents’ perspectives beyond what they pointed out explicitly as having learned. This is suggested by the fact that several identified themes resonate with the main messages in the YAM and KRAS materials. Examples are the importance of seeking help with serious mental health concerns, the potential of influencing your mental health, and that NSSI and mental ill-health are not uncommon but can often be hidden.

### Strengths and Limitations

This study was carried out by a research group with broad research experience, and with extensive knowledge and experience in a range of fields including developmental psychology, educational psychology and child and adolescent psychiatry.

While mental health interventions are mainly evaluated quantitatively to establish evidence for effective treatment, qualitative studies such as the present one have the strengths of being able to answer “how”-type questions, as well as shedding light on the plurality of experiences present in a diverse sample.

The composition of the interview groups can be seen as both a strength and a limitation. As the interviews were conducted with several groups including both boys and girls, and from several schools, the resulting study material included variation with many different voices. Since groups were assembled from a population of adolescents who had given consent to study participation as well as received such consent from their caregivers, all student voices were not included in the present study material. It is possible, for example, that adolescents and their caregivers who gave consent had a special interest in the topic of mental health. Also, adolescents who were not in school, due to truancy, severe physical or mental health problems or other reasons for absence, have not been reached with the current study design.

A methodological strength is that the interview guide included questions and probes ranging from broad to specific, giving rise to material including both free reflection on topics of interest and answers to predefined questions.

Further, a strength associated with the group format is that a larger number of adolescents could be included in the material while keeping the total interview time within reasonable limits. However, it must be assumed that the group setting influenced what could be expressed and how it was said, which is a limitation compared to individual interviews. The setting of the interviews on the school premises and being held by adults in a professional capacity is also something that reasonably shapes the conversation compared to if adolescents were discussing these topics without the presence of adults in other settings.

## Conclusions and Recommendations

A key result is that adolescents generally perceive information on mental health and NSSI in school as needed and important. Further, academic pressure and social media were domains the adolescents highlighted as extra important, and aspects that need to be included when addressing mental health and NSSI.

As the dynamic between autonomy and help-seeking was underscored by the participants, a key implication of the present study is to take adolescents’ need for autonomy into account when setting up student mental health interventions. Adolescents’ need for independence and feeling competent need to be acknowledged and balanced against recommendations about help-seeking.

More research is needed regarding how universal mental health preventive interventions can be tailored to suit a wide range of individual adolescents and how other variables such as group size and setting can be optimized.

Concerning the timing of the intervention and whether interventions should be universal or targeted, adolescents in the current study identified pros and cons, but favored earlier rather than later interventions.

## Electronic supplementary material

Below is the link to the electronic supplementary material.

Supplementary Material 1

## Data Availability

Due to the formulations of the informed consent form, study data cannot be made publicly available. Data are however available from the corresponding author upon reasonable request subject to ethical permissions and participant consent.

## Footnotes

1 The term mental ill-health is used here as it is the best-fitting translation of the Swedish term “psykisk ohälsa”. It should be read not as synonymous only with mental disorder or illness but as a wider term encompassing various degrees of mental health issues including “feeling bad”.

## Abbreviations

NSSI: Nonsuicidal Self-Injury
WHO: World Health Organization
YAM: Youth Aware of Mental Health
SRQR: Standards for Reporting Qualitative
TA: Thematic Analysis
