# Supplementary material for: Adolescents’ experiences of a whole-school preventive intervention addressing mental health and nonsuicidal self-injury: a qualitative study
Source: BMC Public Health. 2024 Dec 2;24:3350. doi: 10.1186/s12889-024-20832-y (PMC11610215; doi:10.1186/s12889-024-20832-y)
Supplement: Supplementary file 1 — Supplementary Material 1 [file 12889_2024_20832_MOESM1_ESM.pdf]

**Interview guide (translated from Swedish)**
**Main and follow-up questions.**

- What were you told in advance about the project at your school? What were your expectations?
- What do you remember from the intervention, from what we did?
  - (Prompts: Lectures, dilemma cards, role-playing, summary, film on nonsuicidal self-injury)
- Did you perceive any main messages?
- What did you take away from the intervention?
  - About mental health?
  - About seeking help?

- What are your thoughts about the project?
  - Do you think an intervention like this can be a good way to prevent mental ill-health?
- Does it concern topics that are important to adolescents? If so, in what way?
- What do you think about working with mental health in the classroom in this way?
  - Was it like you had expected?
  - Pros/cons?
  - Were some things easier/harder? Were there some things you appreciated especially?
- What do you think about the whole-school intervention, trying to reach everyone in the school: students, staff and parents?
  - Do you think that it is helpful that also parents and teachers receive education and information?
- Do you think that the intervention had any impact on students, parents and staff?
  - Did anything change?
- Could the intervention have been delivered in another way? If so, how?
  - Should some things be added/removed?
  - Is there anything that school staff and parents should know more about?
  - Is this ($7^{th}$ and $8^{th}$ grade) the right time to address questions on mental health?

- Did anything change at your school through this project?
  - Are students, staff or parents doing anything differently now?
- Do you think that this kind of project can lead to any changes? In adolescents, staff, or parents? If so, how?
  - Could this have been achieved in another way?
- What effect can this type of project have, do you think?
  - How you think about mental health?
  - How you talk to each other?
  - How you interact with each other?
  - How you seek help and support from others?
  - How you support others?
- Can you think of other changes that could be important?
  - How could such changes be achieved?
